# Lipopeptides efficiently disperse spore crystal aggregates in *Bacillus thuringiensis israelensis*

**DOI:** 10.1007/s00253-026-13901-2

**Published:** 2026-06-23

**Authors:** Maissa Chakroun, Sameh Sellami, Dalel Ben Farhat-Touzri, Nay El Khoury, Hadi Loutfi, Mireille Kallassy Awad, Souad Rouis

**Affiliations:** 1 Centre of Biotechnology of Sfax, P.O. Box ‘‘1177’’ 3018, Sfax, Tunisia; 2 Laboratory of Biodiversity and Functional Genomics, UR-EGP, Université Saint-Joseph de Beyrouth, Beirut, Lebanon; 3 Physics Department, UR TVA, Faculty of Science, Université Saint-Joseph de Beyrouth, Beirut, Lebanon; 4 OPTIMAG Laboratory, IBSAM, University of Western Brittany (UBO), 6 Avenue Le Gorgeu, C.S. 93837, 29238 Brest Cedex 3, France

**Keywords:** *Bt israelensis*, Crystal spore aggregation, Lipopeptide

## Abstract

The separation of *Bacillus thuringiensis* (*Bt*) spores and crystals is often compromised by dense aggregates, particularly in *Bt *subsp. *israelensis*. This study evaluates (sonication), chemical (SDS), and biological (lipopeptide) treatments to improve aggregate dissociation in BUPM98, using AR23 as a reference and Lip (*Bt *subsp. *kurstaki*) as a naturally dissociated control. Initial hexane-based purification highlighted the impact of aggregation, with significantly lower recovery rates for *israelensis* compared to *kurstaki*. Electron microscopic analysis revealed tightly integrated spore crystal complexes in *Bt israelensis* and independent bipyramidal crystals in Lip. While physical sonication and chemical SDS treatments improved dissociation, biological treatment with lipopeptides yielded the most significant results. This approach drastically increased viable spore counts by up to 8000-fold and enhanced crystal recovery to 60%, offering a superior and more efficient biosurfactant-based alternative to traditional methods. PCR analysis identified the kurstakin synthetase gene exclusively in the naturally dissociated *kurstaki* strain, suggesting a native role for these biosurfactants in preventing aggregation. To our knowledge, this is the first demonstration that lipopeptides can effectively dissociate *Bacillus thuringiensis* spore crystal aggregates, offering an efficient biosurfactant-based alternative to physical or chemical methods.

## Introduction

*Bacillus thuringiensis* (*Bt*) is a spore-forming bacterium that synthesizes parasporal crystals composed of protein molecules called delta-endotoxins, which are toxic against the larvae of a wide range of insect orders (Palma et al. 2014; Sellami et al. 2015, 2016). The combination of spore persistence and crystal toxicity makes *Bt* an effective, environmentally safe biocontrol agent widely used in agriculture and public health. The most effective commercial formulations are based on mixtures of spores and parasporal crystals derived predominantly from *Bt *subsp. *kurstaki* and *Bt *subsp. *israelensis*. In fact, *Bt *subsp. *kurstaki* is highly active against Lepidopteran pests, such as caterpillars and moth larvae, making it widely used in crop protection (Sellami et al. 2013; Jamoussi et al. 2013) whereas *Bt *subsp. *israelensis* targets Dipteran larvae, including mosquito and blackfly larvae, playing a crucial role in public health programs (Kaur et al. 2000; De Maagd 2015; Bravo et al. 2011).

The bioinsecticidal activity of these products largely depends on the ratio of spores to delta-endotoxins. Spores ensure environmental persistence, while the crystal proteins provide immediate toxic activity upon ingestion. Consequently, precise quantification of Cry proteins and accurate enumeration of viable spores are critical control parameters during *Bt* fermentation, guiding both process optimization and formulation consistency. Currently, colony-forming unit (CFU) counting remains the standard method for assessing viable spores, while various physical and chemical techniques have been developed for crystal purification. These include gradient centrifugation using sodium bromide, sucrose, or Ludox, and biphasic separation using organic solvents like hexane (Ang and Nickerson 1978; Takebe et al. 2005; Zhu et al. 1989; Rolle et al., 2013; Mounsef et al. 2014). However, crystal yields and purity vary significantly across these protocols and among different *Bt* subspecies, reflecting the inherent diversity in crystal properties and their interaction with spores (Ang and Nickerson 1978; Mounsef et al. 2014).

Additional variability is also observed between cultures grown in solid versus liquid media. Purification from solid medium is more challenging because spores and crystals tend to aggregate forming clusters that are difficult to separate efficiently (Sharpe et al. 1975; 1979). Differences in crystal morphology, surface properties, and the presence of extracellular substances further contribute to variability in yield and purity across methods and strains. Additionally, *Bacillus* spores also adhere to each other and to solid surfaces; the factors responsible for these interactions are mainly physicochemical (Mamane-Gravetz and Linden 2005), van der Waals binding, electrostatic interaction energies, and most importantly, the surface hydrophobicity. These interactions are known to play a major role in the nutrient acquisition (Karasowska and Sigler 2014).

Some *Bacillus* strains produce bio-surfactants called lipopeptides which are amphipathic molecules consisting of both hydrophobic and hydrophilic moieties that partition preferentially at the interface between fluid phases having different degrees of polarity and hydrogen bonding. These molecules are able to modify the surface hydrophobicity of the produced cells and thus their adhesion ability. In fact, *Bt* can produce a lipopeptide called *kurstakin* in reference to its strain of origin *Bt *subsp. *kurstaki* HD1 (Hathout et al. 2000). Unlike the common *Bacillus* produced lipopeptides, *kurstakin* is not recovered in the culture supernatant of producing strains; it is found in association with the spore and is thought to be secreted molecule with high affinity to membrane (Béchetet al. 2012).

This study aimed to overcome crystal aggregation in *Bacillus thuringiensis *subsp.* israelensis* BUPM98 during organic solvent-based purification. We tested sonication, alone or with SDS, and evaluated lipopeptides as a biological additive. This approach significantly improves crystal recovery and effectively overcomes the persistent spore crystal aggregation that limits purification of *Bt *subsp. *israelensis*.

## Material and methods

### Purification of Bt crystals using hexane-based hydrophobic separation

The bacterial strains used in this study included *Bt *subsp.* kurstaki* (Lip and HD1) and *Bt *subsp.* israelensis* (BUPM98 and AR23). Strain BUPM98 was isolated in our laboratory in Tunisia (Zghal et al., 2018), while strains Lip and AR23 were isolated in Lebanon (El Khoury et al. 2014; Fayad et al. 2019). The reference strain HD1 was obtained from a widely available laboratory collection.

Crystal purification was performed via hydrophobic-based separation according to the protocol of Mounsef et al. 2014 with minor modifications. Three *Bt* strains BUPM98, AR23, and Lip were grown on solid T3 sporulation medium (Travers et al. 1987) at 30 °C until completed sporulation. Two grams of freshly harvested biomass were suspended in 20 mL of distilled water and centrifuged for 20 min at 6000 rpm. The obtained pellet was washed three time with 1 M NaCl then suspended in distilled water. Hexane was added to a proportion of 14% (v/v) by sliding it along the tube. The suspension was sonicated in a water bath sonicator (J.P. SELECTA, 200 W, 50/60 Hz) for 10 min to ensure gentle and homogeneous dispersion of spore crystal aggregates while preserving crystal integrity, then centrifuged for 20 min at 6000 rpm. The obtained pellet was resuspended in water and the organic solvent was added again, this procedure was repeated until the optimal crystal purity was reached. Crystal purity was defined as the predominance of crystals with no visible spores or cellular debris as determined by light microscopy. Crystal purity was determined by microscopic examination. After purification, samples were observed under phase-contrast microscopy, and counts of crystals and residual spores were performed in multiple representative fields. Crystal purity was calculated as the percentage of crystals relative to the total number of particles (crystals + spores).

Finally, the final pellet was washed with distilled water and analyzed for the final spore count and the Cry protein concentration. For Cry protein quantification, a part of the final pellet was solubilized in 0.5 M NaOH for 2 h and then centrifuged. The solubilized Cry protein in the supernatant was quantified by the Bradford method (Bradford 1976).

### Purification of Bt crystals using sonication and SDS

Two grams of sporulated *Bt* biomass were suspended in 20 mL of distilled water and sonicated for 2 min using pulse cycles of 20 s on and 20 s off at 240 W and 20 kHz with a Vibra-Cell sonication processor (SONICS) to efficiently disrupt strongly bound spore crystal aggregates. The resulting homogenate was centrifuged at 6000 rpm for 20 min. After that, the pellet was resuspended in 20 mL of a 2% (w/v) SDS solution and incubated for 2 h at 37 °C with continuous shaking. Following incubation, the biomass was collected by centrifugation at 6000 rpm for 20 min. The supernatant was discarded, and crystal purification was completed using the hexane-based separation protocol described in the previous section. The concentration of sodium dodecyl sulfate (SDS) used in this study was chosen according to previously reported experimental conditions and standard protocols, as SDS is a standard synthetic anionic surfactant commonly used as a reference in surfactant-based studies due to its well-characterized and reproducible properties (Kim et al. 2022).

### Lipopeptide-mediated purification of Bt crystals

*Bacillus amyloliquefaciens* strain CEIZ-11 was used for lipopeptide production, extraction, and purification, following the method described by Zouari et al. (2016) who chemically characterized the lipopeptide profile of *B. amyloliquefaciens* CEIZ-11 using RP-HPLC and LC-MS, confirming the presence of surfactin-, iturin-, and fengycin-like families. This ensured that the lipopeptide fraction used in the present study corresponds to a well-defined and previously validated biosurfactant mixture. Briefly, the crude culture broth of strain CEIZ-11 was centrifuged to remove bacterial cells, and lipopeptide present in the cell-free supernatant was precipitated by acidification with HCl. The resulting precipitate was extracted with methanol, after which the solvent was evaporated and the crude lipopeptide extract was resuspended in Tris-HCl buffer.

For crystal purification, completely sporulated *Bt* biomass (2 g) was suspended in 20 mL of the purified lipopeptide solution (10 mg mL^−1^ final concentration) and incubated for 30 min at 30 °C. The resulting suspension was centrifuged at 6000 rpm for 20 min, and the supernatant was discarded. The pellet was washed three times with 1 mol L⁻^1^ NaCl to remove nonspecifically bound components and subsequently resuspended in 20 mL of lipopeptide solution (10 mg mL⁻^1^). Hexane extraction was then performed as described above to complete crystal purification. The concentration of lipopeptides was selected based on previously published studies and was set above the range reported in the literature to ensure sufficient surfactant activity, promoting surface tension reduction and effective dispersion of spore crystal aggregates (Zouari et al. 2016).

### Estimation of Bt spore count

Samples were first heat treated at 98 °C for 10 min to eliminate vegetative cells. Spore counts were determined by enumerating colony-forming units (CFUs) grown on solid LB agar following overnight incubation at 30 °C. For each dilution, counts were performed in four to seven replicates. Results were expressed as mean ± SE (CFU mL⁻^1^). CFU data were analyzed and graphically represented on a logarithmic scale using GraphPad Prism software. Statistical analysis was performed using two-way analysis of variance (ANOVA), followed by Sidak’s post-hoc test to compare spore yields between treatments. Asterisks (^*^ and ^****^) denote statistical significance at *p* < 0.05 and *p* < 0.0001, respectively.

### DNA extraction and PCR detection of kurstakin gene

The genomic DNA of the selected strains Lip, HD1, AR23, and BUPM98 was extracted as described by Magarvey et al. (2004). The quantity and purity of the DNA were measured by the NanoDrop analyzer of Thermo Scientific. PCR reactions were carried out using the following primers: Aks-F (TCHACWGGRAATCCAAAGGG) and Tks-R(CCACCDKTCAAAKAARKWATC) (Abdelrahmani et al. 2011). The PCR mixture was prepared in a total reaction volume of 25 µL containing 2 µL (50 ng) of genomic DNA, 20 mmol L⁻^1^ Tris-HCl, 2.5 mmol L⁻^1^ MgCl₂, 0.2 mmol L⁻^1^ of each of the four dNTPs, 2 µmol L⁻^1^ of each primer, and 2.5 U of Taq DNA polymerase (Amersham). Amplification was performed by an automatic Thermocycler (GeneAmp PCR system 2700, Applied Biosystems) programmed for an initial denaturation at 94 °C for 3 min, followed by 30 cycles of 94 °C for 1 min, an annealing step of 40 s at 44.4°C followed by the extension step for 2 min at 72°C. Final extension was performed at 72 °C for 10 min. The amplified products were also visualized in a 1% agarose gel stained by ethidium bromide. PCR was considered positive when a band of the appropriate size of 1.1 kb was visualized on the agarose gel.

### Scanning electron microscopy (SEM) image acquisition

To ensure consistent particle concentration for SEM analysis, 100 μL of the *Bt* stock solution was diluted in 1.9 mL of distilled water. The mixture was sonicated for 5 min (37 kHz) and vortexed to disperse aggregates. A 50-μL aliquot was then deposited onto cleaved Mica and dried at 37°C. To ensure electrical conductivity, the samples were metallized with a 2.5-nm gold–palladium layer. Finally, imaging was performed using a Hitachi S3200N scanning electron microscope at an accelerating voltage of 15 kV and a working distance of 4 mm (Loutfi et al. 2020).

## Results

### Differential spore crystal separation efficiency in Bt subsp. israelensis (BUPM98 and AR23) and Bt subsp. kurstaki (Lip)

**Fig. 1:**
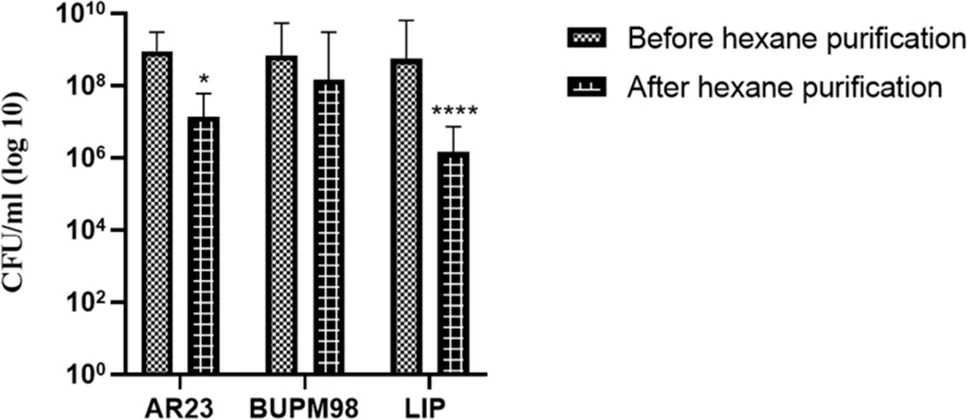
Comparison of spore counts for *Bt *subsp. *israelensis* (AR23 and BUPM98) and *Bt *subsp. *kurstaki* (Lip) before (  ) and after (  ) hexane purification. Values are the mean of four to seven replicates. The data are represented as mean ± SE in a logarithmic scale and statistics were performed using two-way ANOVA with Sidak’s post-hoc test to compare spore yields. ^*^ and ^****^ denote *p* < 0.05 and *p* < 0.0001, respectively

Parasporal crystals from *Bt* strains BUPM98, AR23, and Lip were purified following the method of Mounsef et al. (2014). The purity of the final crystal preparation was assessed by spore counting and by microscopic observation using both optical and electron microscopy. Before hexane-based purification, spore concentrations were quantified by colony-forming unit (CFU) in order to determine the initial level of spores present in each strain. As shown in Fig. 1, the CFU values ranged between approximately 2.0 × 10^9^ and 6.0 × 10^9^ CFU, depending on the strain. The strain *Bt *subsp. *israelensis* BUPM98 exhibited the highest spore concentration compared to AR23, used as a reference strain for *Bt *subsp. *israelensis*, showing lower CFU values, whereas Lip, used as a control strain belonging to *Bt *subsp. *kurstaki*, presented a distinct spore profile.

The purification process started with comparable spore numbers in the homogenates of the three strains. After purification, the number of residual spores in the purified crystal preparations was markedly lower in the *Bt *subsp. *kurstaki* strain compared to the *Bt *subsp. *israelensis* strains. Spore counts in the purified crystal preparation of the *Bt *subsp. *kurstaki* strain Lip decreased by approximately 200-fold after two successive hexane extractions (Fig. 1), resulting in crystal preparations with up to 98% purity (Table 1). In contrast to the *Bt *subsp. *kurstaki* strain, the spore counts in the purified crystal preparations of the *Bt *subsp. *israelensis* strains remained significantly higher. Following five to six successive hexane extractions, the spore concentrations for BUPM98 and AR23 decreased by only 4-fold and 16-fold, respectively (Fig. 1). Besides, the crystal preparation did not exceed 30% and 25% of purity for BUPM98 and AR23, respectively (Table 1).

**Table 1:** Percentage of Cry protein total yield after purification. Data are shown as mean of two replicates ± SEM

	No treatment	Sonication	Sonication + SDS	Lipopeptide
Lip	98.3 ± 0.2	98.4 ± 0.3	99.1 ± 0.1	96.9 ± 0.2
BUPM98	30.5 ± 2.5	34.3 ± 0.3	41.3 ± 6.2	60.2 ± 1.0
AR23	24.8 ± 0.8	33.5 ± 0.5	35.5 ± 1.5	59 ± 0.4

**Fig. 2:**
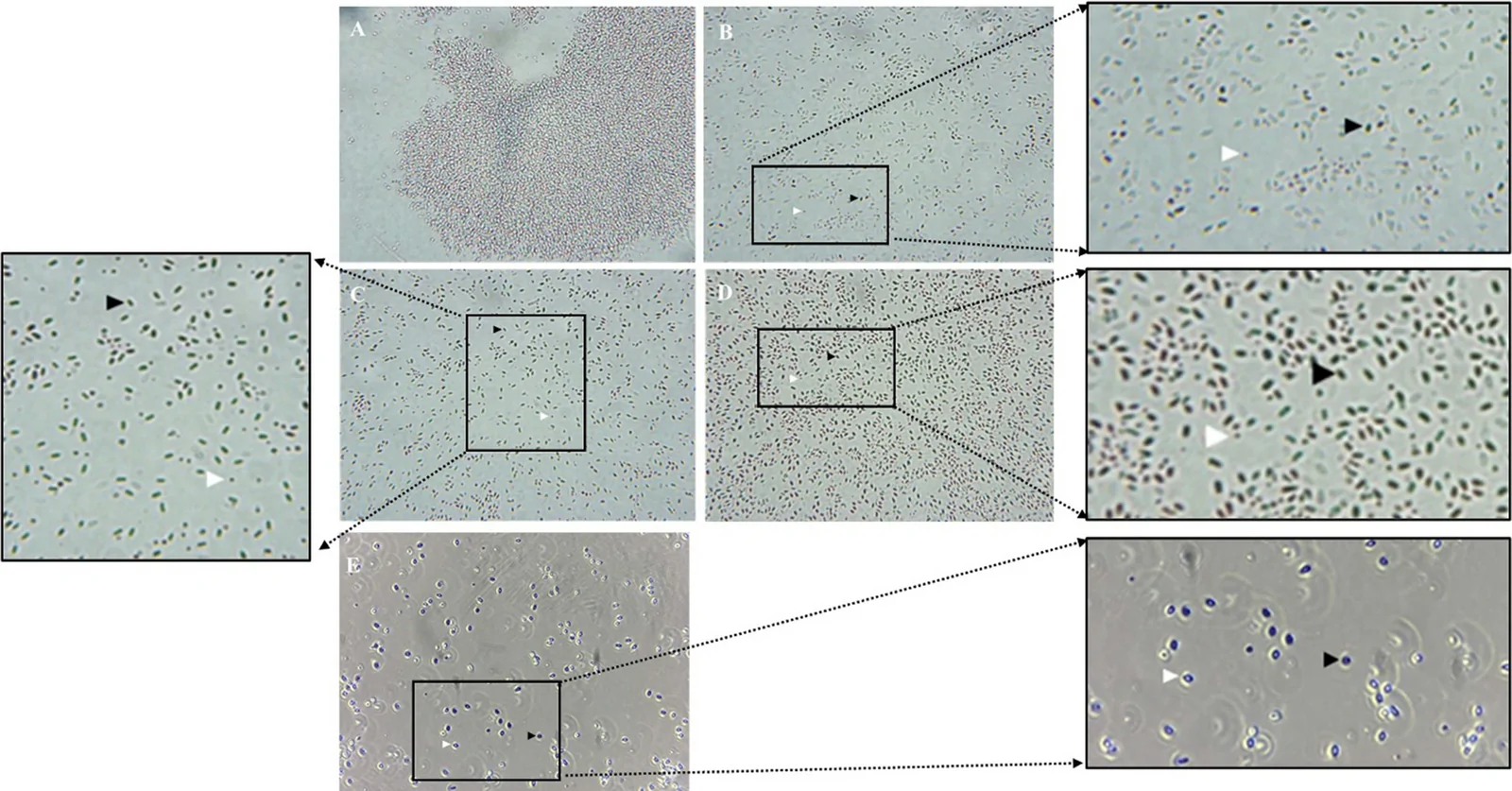
Light microscopic observation of bacterial homogenates. *Bt *subsp. *israelensis* BUPM98 homogenates: **A** untreated control, **B** after sonication, C following sonication and incubation with SDS, and **D** after incubation with lipopeptides. **E** Untreated *Bt *subsp. *kurstaki* Lip homogenate was used as a comparative control. White arrowheads indicate **B**–**D** the small spherical crystals of *Bt *subsp. *israelensis* and **E** the bipyramidal crystals of *Bt *subsp. *kurstaki*. Black arrowheads indicate the spores

**Fig. 3:**
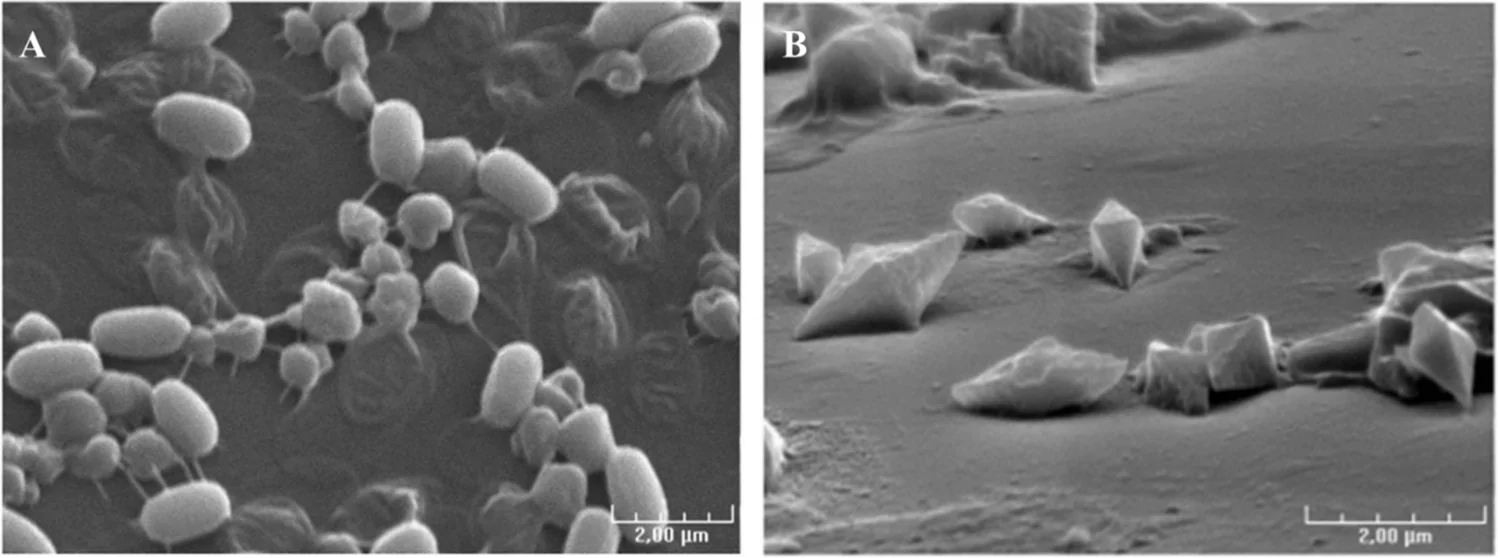
Scanning electron microscopy (SEM) analysis of spore crystal complexes from reference strains *Bt *subsp. *israelensis* (AR23) and *Bt *subsp. *kurstaki* (Lip)

On the other hand, microscopic analysis of the homogenates via light microscopy revealed large spore-crystal aggregates in the *Bt *subsp. *israelensis* strains, whereas these complexes were entirely absent in the *Bt *subsp. *kurstaki* strains (Fig. 2A). These observations were further confirmed by electron microscopy (Fig. 3A and B). In fact, in the *Bt *subsp. *israelensis* strain (Fig. 3A), the spores and crystals appear as integrated complexes, indicating a strong physical attachment that prevents their dissociation. Conversely, the *Bt *subsp. *kurstaki* strain (Fig. 3B) displays independent, bipyramidal crystals entirely separated from the spores.

### Enhancement of crystal recovery and CFU counts in Bt subsp. israelensis strains through combined sonication and SDS treatment

**Fig. 4:**
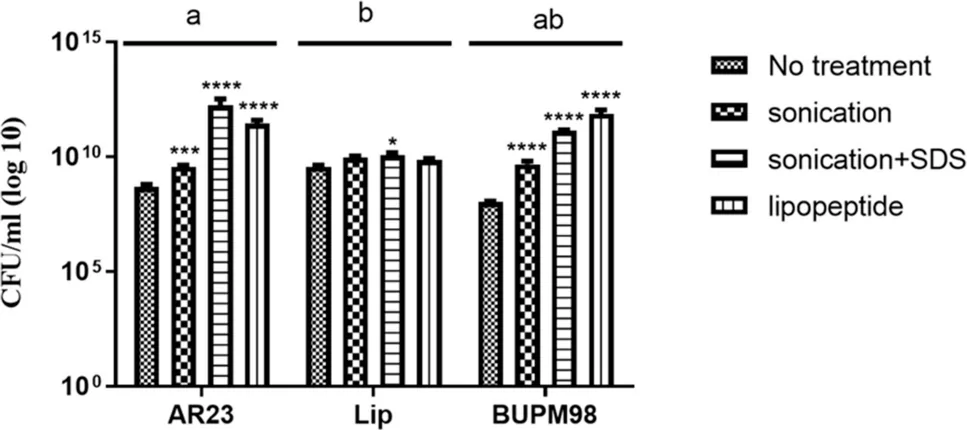
Effect of sonication, SDS, and lipopetide treatments on the spore counts in the homogenate of three *B. thuringiensis* strains. No treatment (  ), sonication (  ), sonication and incubation with SDS (  ), or after incubation with lipopeptide (  ). Values are the mean of three to five replicates. The data are represented as mean ± SE in a logarithmic scale and statistics were performed using two-way ANOVA with Tukey post hoc test to compare spore yields among treatments. ^*^, ^***^, and ^****^ denote *p* < 0.05, *p* < 0.001, and *p *< 0.0001, respectively. Letters denote statistical differences

In order to dissociate the spore crystal aggregates characteristic of *Bt *subsp. *israelensis* strains, homogenate of BUPM98 and AR23 were sonicated with Lip serving as a naturally dissociated control. Light microscopy of the sonicated BUPM98 homogenate revealed a marked reduction in aggregate size (Fig. 2B). Furthermore, sonication led to an increase in colony-forming units (CFU) by 50-fold, 7-fold, and 2.5-fold for BUPM98, AR23, and Lip, respectively (Fig. 4). In addition, a measurable improvement in crystal yield was observed for both *Bt *subsp. *israelensis* strains (Table 1). Specifically, the yield for BUPM98 increased from 30 to 34.3% following sonication compared to AR23 from 25 to 33.3%. In contrast, the *Bt *subsp. *kurstaki* strain (Lip) maintained a nearly perfect yield of 98%. Since sonication alone provided only a moderate increase in crystal recovery, we aimed to further dissociate spore-crystal aggregates using SDS as a chemical additive. The combination of sonication and SDS significantly reduced the number and size of microscopic aggregates (Fig. 2C), leading to CFU increases of 1000-fold and 5000-fold for BUPM98 and AR23, respectively, and 3-fold for Lip, whose aggregates are naturally dissociated (Fig. 4). In addition, the sonication and SDS combination improved the crystal yield to 41% for BUPM98 and 35.5% for AR23 (Table 1).

### Enhancement of spore crystal dispersion using lipopeptides

Physical treatment by sonication and chemical treatment with SDS provided only moderate improvements for the dissociation of *Bt *subsp. *israelensis* spore crystal aggregate. For this reason, lipopeptides were used as biosurfactants to further improve the dissociation rate by reducing surface tension and disrupting hydrophobic interactions. Light microscopic observation showed almost complete disappearance of the aggregates for BUPM98 (Fig. 2D). CFU analysis showed an 8000-fold and 600-fold increase for BUPM98 and AR23, respectively, while Lip exhibited only a minor 2-fold increase, reflecting its naturally dissociated state (Fig. 4). Correspondingly, the crystal recovery yield increased significantly for BUPM98 to 60% and for AR23 to 59%, whereas no substantial change was observed for Lip, confirming that its crystals are already efficiently purified without treatment (Table 1).

### PCR detection of kurstakin synthetase gene

**Fig. 5:**
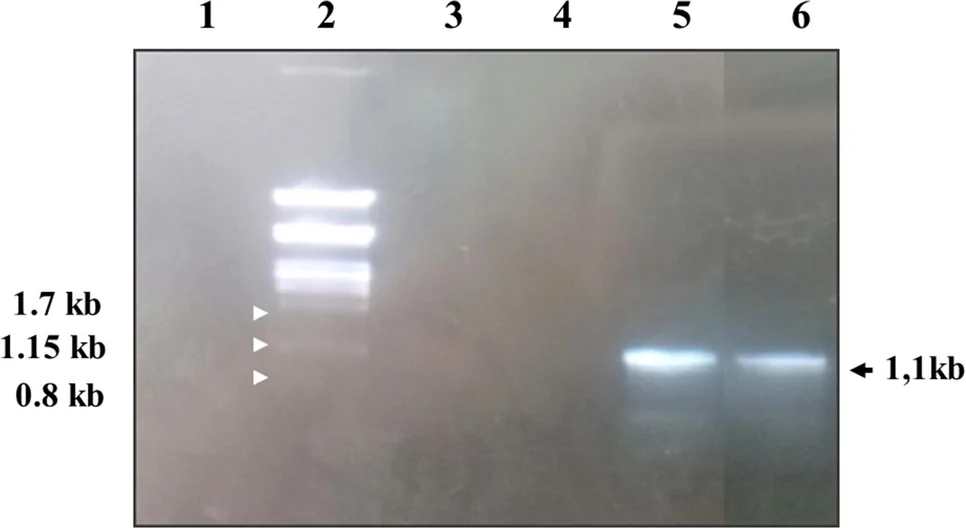
*Kurstakin* gene detection by PCR. Lane 1: negative control. Lane 2: molecular weight marker (11.5, 5, 4.7, 4.5, 2.8, 2.5, 2.4, 2.1, 1.9, 1.7, 1.15, 1, 0.805, and 0.514 kb). Genomic DNA from: Lane 3: BUPM98; Lane 4: AR23; Lane 5: HD1; and Lane 6: Lip

In order to determine whether the gene encoding for *kurstakin synthetase* is present in the *Bt* strains, PCR amplification was performed using *kurstakin synthetase* gene with the specific primers Aks-F and Tks-R. The positive control strain HD-1, as well as Lip strain produced an amplicon of approximately 1.1 kb corresponding to the target gene. BUPM98 and AR23 showed no amplification for the *kurstakin synthetase* gene, confirming the absence of such gene in these strains (Fig. 5).

## Discussion

*Bt* crystal purification is a key step for the study of their microbiological and biochemical properties as well as their mode of action and toxicity independent of spore. Several techniques have been developed for this purpose (Pendelton and Morisson 1966; Goodman et al. 1967; Fast and Morrison 1972; Sharpe et al. 1975; Milne et al. 1977; Ang and Nikerson 1978; Sharpe et al. 1979; Mahillon and Delcour 1984; Murtyet al. 1994; Mounsef et al. 2014). The efficacy of these techniques and the crystal recovery yield were very different from a strain to another; however, no explanation of the possible causes of these differences was evidenced (Goodman et al. 1967; Ang and Nickerson 1978; Mahillon and Delcour 1984; Murtyet al. 1994; Mounsef et al. 2014). In this study, we examined for the first time the possible causes of the difference in the crystal recovery yield between strains from *Bt *subsp. *israelensis* and *Bt *subsp. *kurstaki*.

The purification technique used in this work is based on the hydrophobic characteristic of *Bacillus* spores; the most hydrophobic spore has the greater affinity toward hexane hydrophobic surface (Rosenberg et al. 1980; Doyle et al. 1984; Koshikawa et al. 1989). In the same way, a spore can provide a surface of attachment to another spore leading to the aggregation (Ronner et al. 1990; Faille et al. 2002; Mamane-Gravetz and Linden 2005). In the case of *Bt *subsp. *israelensis*, the aggregating spores are also entrapping the crystals giving rise to macroscopic particles which are preventing the spores from floating to the hexane hydrophobic surface and be separated from the crystals leading most probably to the low crystal yield at the end of the purification. It was also reported that filamentous appendages which are structures arising from the exosporium of some *Bacillus* species including *Bt* may contribute to their hydrophobicity (Hachisuka et al. 1984; Koshikawa et al. 1989). Sonication or incubation with SDS of *Bacillus* spores reduces their hydrophobicity, respectively, by removing part of the exosporium and breaking the filamentous appendages (Escobar-Cortés et al. 2013; Thompson et al. 2011) and by dissociation the spore adsorbed proteins (Beamanet al. 1971; Kozuka and Tochikubo 1985; Thompson et al. 2011; Redmond et al. 2004). The effect of sonication either alone or in combination with the SDS was tested on the spore/crystal homogenate of BUPM98 and AR23. CFU of the sonicated homogenate was lower than the CFU of the sonicated and incubated with SDS, which means that the SDS is enhancing the effect of sonication for the spore disaggregation. Positive correlation was observed between the CFU increase in the sonicated and SDS-treated homogenate and BUPM98 crystal yield improvement, this observation confirms our hypothesis of the crystals sequestration by the spore aggregates. In fact, when the aggregates were disrupted the spores and crystals were dissociated; the former could join the hexane hydrophobic interface and be separated from the latter. For AR23, despite the significant increase in CFU following SDS treatment, no improvement in crystal yield was observed. This suggests that while SDS effectively disperses spores, it may partially destabilize AR23 crystals or alter their hydrophobic interactions, preventing efficient separation and limiting recovery.

Lip has no visible aggregates under microscope, their CFU was not significantly affected neither by the sonication nor the SDS, and their crystal recovery yield at the end of the purification was not affected too. Bringing all these data together suggest that the spores of the *kurstaki* strains have a particular characteristic that allow them to stay dissociated in suspension without forming aggregates. Hathout et al., in 2000, isolated a new class of lipopeptides from *Bt *subsp. *kurstaki* HD-1 he called *kurstakin*. In fact, lipopeptides are known to modify the local surface tension to facilitate the invasive growth of the producing strains (Barakatet al. 2017), *kurstakin* particular was not secreted to the culture medium, but is found tightly associated to the bacterial membrane and particularly to the spore (Hathout et al. 2000; Price et al. 2007; Abderrahmani et al. 2011). The addition of amphiphilic lipopeptides triggers a rapid physicochemical modification that dissociates the *Bacillus* spore crystal complex. This process is driven by the intercalation of hydrophobic fatty acid chains between the entities, disrupting their initial hydrophobic adhesion (Markelova et al., 2025; Kumari et al. 2023). A concomitant reduction in interfacial tension allows the aqueous medium to penetrate the interface, while lipopeptide adsorption masks surface hydrophobicity by orienting hydrophilic peptide heads away from the surface. This creates an entropic and electrostatic barrier that prevents reaggregation and ensures stable physical separation (Hsu et al. 2025; Kourmentza et al., 2021). The synergistic action of surfactin, iturin, and fengycin, purified and characterized by Zouari et al. (2016), specifically separates the *israelensis* spore crystal complex. Surfactin drastically lowers tension at the solid–liquid interface, allowing water to penetrate the tight space between the entities. Simultaneously, the iturin and fengycin families intercalate between them, breaking the initial binding force. In contrast to glycolipids such as rhamnolipids, which primarily facilitate overall mixing through emulsions, this lipopeptide mixture ensures clean physical separation while keeping toxin crystals intact and avoiding denaturation risks (Hsu et al. 2025). Accordingly, to explore this hypothesis, lipopeptides Lip and HD1 and absent in BUPM98 and AR23, these results suggest that *kurstakin* produced by Lip strain is tightly associated to the spore surface and is transforming the spore hydrophobic state into a polarized state that allow it to stay dissociated.

To confirm our hypothesis, we added lipopeptides from *B. amyloliquefaciens* CEIZ-11 to the homogenate of *Bt *subsp. *israelensis* strains BUPM98 and AR23. The use of exogenous lipopeptides rather than genetically modified strains was chosen to provide a functional and application-oriented approach, avoiding pleiotropic effects associated with NRPS gene cluster manipulation, while allowing direct evaluation of biosurfactant activity on spore crystal dispersion. The ability of the lipopeptides to dissociate the spore aggregates of these strains was confirmed by microscopic observation and CFU, and the crystal recovery yield was increased two times and was more efficient for Cry purification than sonication and SDS. These results confirm that the lipopeptide produced by *Bt *subsp. *kurstaki* strain is a major factor in the reduction of their spore hydrophobicity and their high purity and crystal recovery yield. In fact, Ben Ayed et al. 2015 showed that lipopeptides from *B. amyloliquefaciens* AN6 had better emulsifier activity than SDS and Tween 80. *Kurstakin* isolated from *Bt* strain 7SA and used as emulsifying agent, was found to increase the hydrophobicity of *Acinetobacter haemolyticus* 2SA strain and improve its diesel oil biodegradation rate (Diallo et al. 2021).

In conclusion, this study highlights the limitations of physical and chemical approaches for disrupting *Bacillus thuringiensis *subsp. *israelensis* spore crystal aggregates. It focuses on functional biochemical validation rather than genetic dissection of lipopeptide biosynthesis, providing a practical and application-oriented strategy for improving crystal recovery in industrial and biocontrol contexts. In contrast, lipopeptide supplementation proved to be an efficient and effective alternative for enhancing crystal purification in non-producing strains. Overall, these findings suggest a promising biosurfactant-based strategy for improving downstream processing of *Bt* formulations.

## Data Availability

All data generated or analysed during this study are included in this published article.
